# Genoa Syndrome and Central Diabetes Insipidus: A Case Report

**DOI:** 10.4274/jcrpe.v2i2.89

**Published:** 2010-05-08

**Authors:** Bülent Hacıhamdioğlu, Zeynep Şıklar, Şenay Savaş Erdeve, Merih Berberoğlu, Gülhiz Deda, Serap Teber Tıraş, Suat Fitöz, Gönül Öçal

**Affiliations:** 1 Department of Pediatric Endocrinology, Ankara University School of Medicine, Ankara, Turkey; 2 Department of Pediatric Neurology, Ankara University School of Medicine, Ankara, Turkey; 3 Department of Radiology, Ankara University School of Medicine, Ankara, Turkey; +90 312 595 67 91+90 312 362 05 81hacihamdi@mynet.comDepartment of Pediatric Endocrinology, Ankara University School of Medicine, Cebeci 6100 Ankara, Turkey

**Keywords:** Genoa syndrome, holoprosencephaly, craniosynostosis, cleft lip/palate, central diabetes insipidus

## Abstract

Genoa syndrome was first described by Camera et al in 1993 in two patients with semilobar holoprosencephaly (HPE), craniosynostosis and abnormal small hands with cone−shaped epiphyses and hypoplastic terminal phalanges of fingers (OMIM: 601370). In 2001, Lapunzina et al reported a case of craniosynostosis and HPE associated with several other malformations and suggested that these findings could be attributed to a severe form of Genoa syndrome or to a newly recognized syndrome. Endocrinopathies in association with HPE are frequently reported in the literature. Diabetes insipidus, hypothyroidism, hypocortisolism, and growth hormone deficiency are frequently associated with HPE. We here report a case of semilobar HPE, craniosynostosis and cleft lip/palate, possibly a case of Genoa syndrome, associated with central diabetes insipidus.

**Conflict of interest:**None declared.

## INTRODUCTION

Genoa syndrome was first described by Camera et al in 1993 (1). They reported two patients with semilobar holoprosencephaly (HPE), craniosynostosis involving the coronal and lambdoid sutures and abnormal small hands with cone−shaped epiphyses and hypoplastic terminal phalanges of fingers. The authors named this possibly autosomal recessive disorder "Genoa syndrome," after the name of the city where both cases were described. In 2001, Lapunzina et al (2) reported a case of craniosynostosis and HPE associated with several other malformations such as cloverleaf skull, Dandy−Walker malformation, bilateral microphthalmia, cleft soft palate, congenital scoliosis. They concluded that these findings could be attributed to a severe form of Genoa syndrome or to a newly recognized syndrome.

HPE is the most common developmental defect of the forebrain and mid−face in humans and occurs in 5−12/10000 live births. Clinically, there is a continuous spectrum of malformations consistent with HPE. Endocrinopathies, such as diabetes insipidus, hypothyroidism, hypocorticism, and growth hormone deficiency, are frequently associated with HPE (3).

We report a case of semilobar HPE, craniosynostosis and cleft lip/palate, considered to be a case of Genoa syndrome, which was associated with central diabetes insipidus.

## CASE REPORT

**Figure 1:**
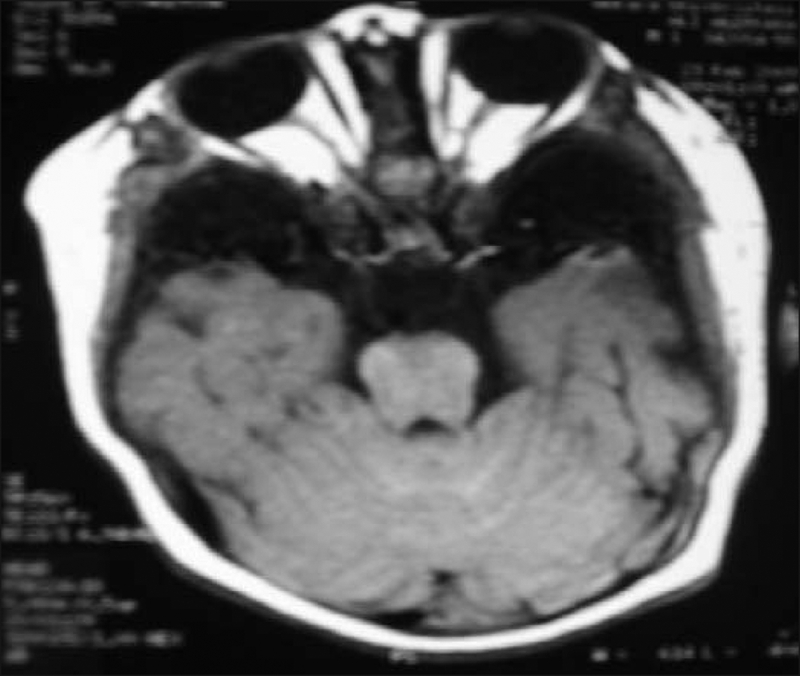
Semilobar HLP

A 12−month−old boy was admitted to our department with hypernatremia. He was born after 40 weeks of gestation as the first child of a first cousin consanguineous couple. There was no family history of congenital defects; the pregnancy was uneventful. Brain magnetic resonance was performed and showed semilobar HPE (Figure 1). The patient was operated for craniosynostosis when he was 8 months old. Fifteen days after the surgery he had seizures, was diagnosed as having West syndrome and started on vigabatrine therapy. His cleft palate was surgically reconstructed at the age of 11 months. The patient did not have any seizures after 10 months of age. During his follow−up, hypernatremia was noted. He was diagnosed with diabetes insipidus and received desmopressin acetate (dDAVP) therapy. He was referred to our department atfer a hospitalization for pneumonia . At presentation, he had been off dDAVP for ten days.

**Figure 2:**
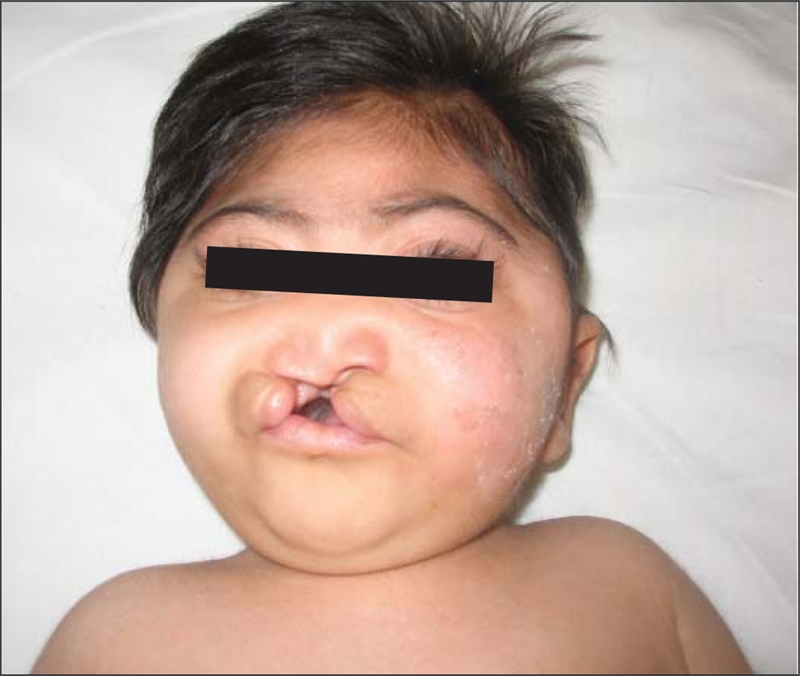
Microcephaly, bilateral cleft lip and flat nose

**Figure 3:**
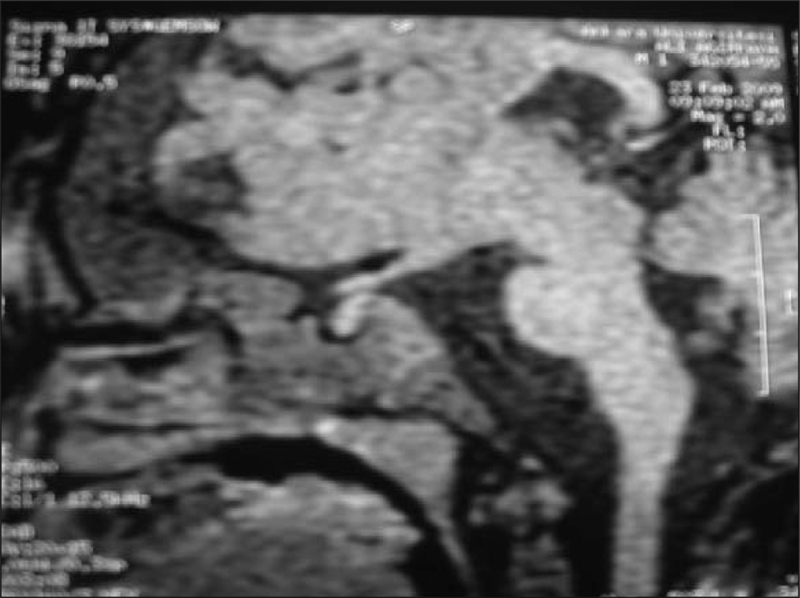
Absence of normal neurohypophysial hyperintensity

On admission, he had microcephaly (40.5 cm <3^rd^ percentile), bilateral cleft lip, a flat nose (Figure 2). His body weight was 6.6 kg (<3^rd^ percentile) and height was 67 cm (10th percentile). There were no clinical signs of dehydration in spite of severe hypernatremia (Na: 161 mEq/L). Serum and urine osmolarity were 337 and 157 mOsm/kg, respectively (urine osmolarity/serum osmolarity ratio: 0.46). Urine specific gravity was 1005; urine sodium was 61 mmol/L. He had polyuria (5.9 mL/kg/h). Serum antidiuretic hormone (ADH) level was 0.7 pmol/L (N:2−8 pmol/L). We re−started dDAVP therapy (2x2.5.μ/bid). Pituitary gland MRI showed absence of normal hyperintensity of neurohypophysis (Figure 3). After dDAVP therapy, serum sodium level decreased to 148 mmol/L, serum osmolarity was normalized (289 mosm/kg/H20) and urine osmolarity was 300 mosm/kg/H20 (urine osmolarity/serum osmolarity ratio: 1.03). His karyotype was normal.

The patient had no clinical or laboratory signs of other pituitary hormone deficiencies. He responded very well to vasopressin treatment with restoration of serum electrolytes, which remained within normal limits at the 6−month follow−up. Clinical response to vasopressin confirmed the diagnosis of central diabetes insipidus.

## DISCUSSION

Syndromes with craniosynostosis have been extensively reviewed elsewhere by Cohen and MacLean (4). To date, there are more than 100 disorders with craniosynostosis as a manifestation. In 1993, Camera et al reported an unusual type of craniosynostosis associated with HPE and several skeletal anomalies in two sibs. This rare type of craniosynostosis was named as Genoa syndrome. In addition to semilobar HPE and craniosynostosis, this case also had craniofacial asymmetry, hydrocephalus, brachycephaly, plagiocephaly, hypotonia, ocular hypotelorism, clinodactyly, hypoplastic terminal phalanx, cone−shaped epiphyses, small vertebral bodies, and slender long bones (1). Lapunzina et al (2) reported a case of craniosynostosis and HPE associated with several other malformations such as cloverleaf skull, Dandy−Walker malformation, bilateral microphthalmia, cleft soft palate, congenital scoliosis, aortic coarctation with patent ductus arteriosus. They concluded that these findings could be attributed to a severe form of Genoa syndrome or to a newly recognized syndrome. Our case had semilobar HPE, bilateral coronal and metopic craniosynostosis, microcephaly, epicanthal fold, flat nose, hypotonia, bilateral cleft lip/palate and growth delay. Contrary to the cases previously described, our patient did not show upslanting palpebral fissures, pigmented retinal degeneration, small hands nor other skeletal anomalies. Also our patients did not have a cardiac anomaly. We evaluated our patient as Genoa syndrome because of semilobar HPE and craniosynostosis, two findings being the main features in patients diagnosed with Genoa syndrome in previous reports.

Because only three cases with Genoa syndrome have been reported so far, additional cases are needed to clarify the several features of this unusual syndromic craniosynostosis and HPE.

HPE is a condition in which the early forebrain does not undergo diverticulation and instead of it develops into a single, unpaired forebrain, the so−called holoprosencephalon. It can be lobar, semilobar, variant, or alobar (with a large holoventricle). In the semilobar HPE, the facial anomalies are mild or absent (5). Seizures are often present. Yoshikawa et al (6) reported the case of a child with West syndrome associated with semilobar HPE. Our patient was also diagnosed as having West syndrome, when he was 8 months old.

Endocrinopathies in association with HPE are frequently reported in the literature. Children with HPE are at risk for hypothalamic and pituitary dysfunction because of midline defects. In a review of 117 children with HPE, Hahn et al (7) identified hypothyrodism in 11%, hypocortisolism in 7%, and growth hormone deficiency in 5% of patients; diabetes insipidus occurred in 70% of patients with classic HPE. This is not surprising, because the hypothalamus is located medially and rostrally in the early fetal maps and therefore is more frequently noncleaved than structures located farther from the midline or more caudally.

Posterior pituitary dysfunctions are more common than anterior ones. Due to the abnormal hypothalamic–infundibular region in HPE, a defect in the supraoptic and paraventricular nuclei of the hypothalamus or in the release of vasopressin via the infundibulum and posterior pituitary can lead to diabetes insipidus. Some authors have attributed the occurrence of diabetes insipidus to abnormal hypothalamic osmoreceptors as normal posterior pituitary is often visualized on MRI, while others hypothesized that the genes involved in the cerebral malformation have secondary genetic effects on the development of the hypothalamic neurons (5). Diabetes insipidus can even be recognized in infancy in patients with HPE (8). Based on the absence of normal neurohypophysis hyperintensity, we believe that diabetes insipidus in our patient was due to an anatomical defect.
